# Caffeine Alters Anaerobic Distribution and Pacing during a 4000-m Cycling Time Trial

**DOI:** 10.1371/journal.pone.0075399

**Published:** 2013-09-18

**Authors:** Ralmony de Alcantara Santos, Maria Augusta Peduti Dal Molin Kiss, Marcos David Silva-Cavalcante, Carlos Rafaell Correia-Oliveira, Romulo Bertuzzi, David John Bishop, Adriano Eduardo Lima-Silva

**Affiliations:** 1 Sports Science Research Group, Department of Physical Education and Sports Science (CAV), Federal University of Pernambuco, Vitoria de Santo Antão, Pernambuco, Brazil; 2 Nephrology Division, Department of Medicine, Federal University of São Paulo, São Paulo, São Paulo, Brazil; 3 Endurance Sports Research Group, School of Physical Education and Sport, University of São Paulo, São Paulo, São Paulo, Brazil; 4 Institute of Sport, Exercise and Active Living (ISEAL), Victoria University, Melbourne, Victoria, Australia; University College London, United Kingdom

## Abstract

The purpose of the present study was to investigate the effects of caffeine ingestion on pacing strategy and energy expenditure during a 4000-m cycling time-trial (TT). Eight recreationally-trained male cyclists volunteered and performed a maximal incremental test and a familiarization test on their first and second visits, respectively. On the third and fourth visits, the participants performed a 4000-m cycling TT after ingesting capsules containing either caffeine (5 mg.kg^−1^ of body weight, CAF) or cellulose (PLA). The tests were applied in a double-blind, randomized, repeated-measures, cross-over design. When compared to PLA, CAF ingestion increased mean power output [219.1±18.6 vs. 232.8±21.4 W; effect size (ES)  = 0.60 (95% CI = 0.05 to 1.16), p = 0.034] and reduced the total time [419±13 vs. 409±12 s; ES = −0.71 (95% CI = −0.09 to −1.13), p = 0.026]. Furthermore, anaerobic contribution during the 2200-, 2400-, and 2600-m intervals was significantly greater in CAF than in PLA (p<0.05). However, the mean anaerobic [64.9±20.1 vs. 57.3±17.5 W] and aerobic [167.9±4.3 vs. 161.8±11.2 W] contributions were similar between conditions (p>0.05). Similarly, there were no significant differences between CAF and PLA for anaerobic work (26363±7361 vs. 23888±6795 J), aerobic work (68709±2118 vs. 67739±3912 J), or total work (95245±8593 vs. 91789±7709 J), respectively. There was no difference for integrated electromyography, blood lactate concentration, heart rate, and ratings of perceived exertion between the conditions. These results suggest that caffeine increases the anaerobic contribution in the middle of the time trial, resulting in enhanced overall performance.

## Introduction

Pacing strategy can be defined as the changes in power output/velocity that occur throughout a time trial (TT) in order to reach the end point in the fastest possible time [1]. Because energy supply during a middle-distance cycling time-trial (e.g. 4000-m TT) is provided by both aerobic and anaerobic pathways, the pacing strategy depends on the momentary rate of energy supply by each of these systems [1]. Nevertheless, as the total amount of anaerobic work generated during a short-distance TT has been considered a fixed and limited amount (i.e. the anaerobic capacity) [2], [3], anaerobic power output distribution has been considered the main metabolic pathway determining both pacing strategy [2], [3] and performance [4] during such events.

It has been suggested that athletes subconsciously monitor some aspects derived from their anaerobic energy expenditure so that near-zero values of the anaerobic reserve are never reached during a TT [2], [3], [5]. This monitoring process was suggested to be based on distance remaining, remaining anaerobic reserves, and momentary power output [5]. However, studies have reported a significant benefit of a fast-start strategy on short-distance cycling TT performance, and this has been associated with a greater anaerobic contribution in the first part of the trial [2]–[4]. For example, Aisbett et al. [4] compared different pacing strategies (fast-, even-, and slow-start) during a cycling TT lasting ∼5 min (approximately the duration of a 4000-m cycling TT) and found that a fast-start strategy was associated with a higher power output and oxygen deficit (indicating greater anaerobic contribution) during the first 25% of the trial, compared with even- and slow-start strategies. The power output and anaerobic contribution during the fast-start trial became lower than the even- and slow-start trials from the second to the last quarter of the trial, resulting in a similar total amount of anaerobic work during the trial, but an increased overall performance. These data reinforce the hypothesis that anaerobic power output distribution is an important factor determining performance. In addition, Craig et al [6] found a significant correlation between anaerobic capacity, measured by maximum accumulated oxygen deficit (MAOD) (i.e. total amount of anaerobic work), and performance during a 4000-m individual pursuit, suggesting that the maximum amount of ATP potentially supplied by the anaerobic energy system may also be an important determinant of performance. This suggests that any intervention able to increase either moment-by-moment anaerobic power output and/or the total amount of anaerobic work may improve performance during a middle-distance cycling TT.

While research suggests the choice of pacing strategy does not affect the total anaerobic contribution [2]–[4], caffeine consumption (from 3 to 9 mg.kg^−1^ body mass) has been reported to increase both the total anaerobic energy contribution and performance during time-to-exhaustion tests [7]–[9]. For example, Bell et al. [7] found an increase in both the MAOD (7%) and time to exhaustion (8%) during high-intensity exercise performed at 125% of the maximal oxygen uptake (VO_2max_) after caffeine ingestion (5 mg.kg^−1^). It is believed that caffeine may increase glycolytic turnover due to an increase in phosphofructokinase (PFK) activity, resulting in an increased rate of ATP resynthesis [10]. Caffeine ingestion also seems to have a direct action on the central nervous system (CNS) [11], which could increase muscle recruitment through the propagation of signals between the motor cortex and motoneurons [12]. Caffeine's ergogenic effects have also been attributed to a blunting of pain [13] and the RPE [11], via blockade of adenosine A_2_a receptor [14].

Similar to time-to-exhaustion tests, improvement in short-distance cycling TT performance has been also found following caffeine ingestion [15]–[17]. Wiles et al [17] found an increase (+3.6%) in the mean power output and a reduction (−3.1%) in time to complete a 1-km cycling TT after caffeine intake (5 mg.kg^−1^ body mass) compared to placebo. However, anaerobic contribution, pacing strategy and muscle recruitment have not been measured in these studies. Therefore, to date, it still is not known if the improved short-distance cycling TT performance caused by caffeine ingestion is due to an increased anaerobic contribution, an increase in muscle recruitment, or both. Furthermore, any changes in anaerobic metabolism would also be expected to affect the work-rate distribution (pacing strategy) during a TT [2]–[4]. Studies investigating the effect of caffeine on pacing strategy, distribution of anaerobic contribution, and muscle recruitment could provide important insights into important physiological mechanisms explaining the ergogenic effect of caffeine on performance.

Therefore, the aim of this study was to investigate the effects of caffeine on pacing strategy, distribution of the anaerobic contribution, and muscle recruitment during a 4000-m cycling TT. We hypothesized that, if caffeine increases the anaerobic energy contribution at any particular point of the trial, it would also modify the pattern of power output distribution during the TT, increasing the total anaerobic contribution and overall performance.

## Methods

### Participants

Eight trained male cyclists volunteered to participate in this study. The sample size required was estimated from the equation 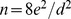, as suggested by Hopkins [18], where n, e, and d denote predicted sample size, coefficient of variation, and the magnitude of the treatment effect, respectively. Coefficient of variation was assumed to be 0.9% [19]. Expecting a magnitude of effect for the treatment of 3.1% [17], detection of a very conservative 1% difference as statistically significant would require at least 6 participants. Considering any possible sample loss, we targeted to recruit 8 participants. The characteristics of participants are described in Table 1. All participants regularly trained ∼223 km.week^−1^, from 5 to 6 times per week, ∼2.5 h per session and had been training for the last ten years without any long interruption (>2 months). Participants were informed about the experimental procedures and signed an informed consent form before the investigation. This study was approved by the Ethics and Research Committee of the Federal University of Alagoas.

**Table 1:** Characteristics of the participants.

	Mean	SD
Age (years)	32.6	5.4
Height (cm)	172.9	4.7
Body mass (kg)	76.7	10.4
Percentage body fat (%)	10.6	4.2
PO_max_ (W)	232	13
VO_2max_ (L.min^−1^)	4.38	0.42
VO_2max_ (mL.kg^−1^.min^−1^)	57.5	5.8
HR_max_ (bpm)	190	4

Values are mean ± SD. PO_max_: maximal power output achieved in the incremental test; VO_2max_: maximal oxygen consumption; HR_max_: maximal heart rate.

### Experimental design

Each participant visited the laboratory on four occasions. On the first visit, participants’ anthropometric measurements were recorded including body mass, height and percentage body fat [20]. Then, the participants performed an incremental test to determine maximal aerobic power (PO_max_) and VO_2max_. On the second visit, the participants performed a 4000-m cycling TT as a familiarization session. On the third and fourth visits, participants performed a 4000-m cycling TT using a double-blind, randomized and repeated-measures crossover design. One hour before starting the experimental session, the participants ingested one capsule containing either 5 mg.kg^−1^ body mass of caffeine (CAF) or cellulose (PLA). Capsules were ingested one hour before the test as this has been described as an adequate time for caffeine digestion and absorption [21]. The treatments were separated by seven days and were conducted at the same time of day (in the morning) in a stable environment in the laboratory (23.0±0.3°C and 44.1±1.3%). Power output (PO), oxygen uptake (VO_2_), electromyography activity (EMG), heart rate (HR), rating of perceived exertion (RPE) and blood lactate concentration [La] were measured during the tests. Participants were informed to refrain from consuming caffeine-containing substances (i.e., coffee, chocolate, and soft drinks) or performing heavy training for 24 h before each experiment. A list of caffeine-containing substances was given to participants before every trial. Participants were also asked to complete a 24-h food record before the first experimental trial and to replicate it in the following experimental trial. Similarity between diets before the trials was checked later by analysis of the writing 24-h food records.

### Incremental test

Each participant performed a maximal incremental test on a cycle simulator (Tacx Flow T1680, Tacx, Wassenaar, Netherlands) that consisted of a 3-min warm-up at a PO corresponding to 100 W, followed by increments of 30 W every 3 min until voluntary exhaustion or when the participants were not able to maintain the pedal frequency between 80–90 revolutions per minute (rpm). During the entire test, breath-by-breath measurements of VO_2_, VCO_2_ and VE were obtained using an automatic gas-exchange analyzer (Quark b^2^, Cosmed, Italy) and subsequently converted to thirty-second averages. The gas analyzer was calibrated before each test using room air and known gas concentrations (16% O_2_ and 5% CO_2_), and the ventilometer with a three-liter syringe (Cosmed, Rome, Italy). VO_2max_ was assumed when at least one of the following criteria was attainted: HR greater than 90% of the maximal age-predicted value; RER greater than 1.10; VO_2_ rate of increase smaller than 150 mL.min^−1^ [22], [23]. The PO_max_ was determined as the highest PO maintained during the last completed stage. When the last stage was not completed, the PO_max_ was calculated from the following equation [24]:
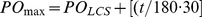



Where: PO_LCS_ is the power output in the last complete stage performed, t is the time in seconds sustained in the last incomplete stage, 180 is the duration of each stage, and 30 is the increment of PO between the stages.

### Familiarization

At least 48 h after the incremental test, the participants performed a familiarization session for the maximal voluntary isometric contractions (MVC) and a 4000-m cycling TT. For the TT, the seat was adjusted vertically and horizontally to each cyclist before the trial, and cycling shoes were used to secure the feet to the pedals. The seat position was recorded and replicated during all subsequent experimental sessions. The participants underwent a warm-up at 100 W for 5min at 90 rpm. All participants started in a fixed gear ratio (i.e., 53×16), but participants were allowed to change gear ratio as desired immediately after the trial had started. Participants were asked to complete the 4000m cycling TT as quickly as possible. During the trial, feedback about the distance covered was provided verbally every 200-m.

### Experimental tests

In the morning of the experimental test, the participants arrived at the laboratory at 0800 h after consuming breakfast between 0700 h and 0715 h. The breakfast was standardized and consisted of 60% carbohydrate (CHO), 25% lipids and 15% protein, without caffeine. One hour before the test, the participants ingested one capsule containing caffeine or placebo, with 150 ml of water. Then, the participants rested for 45min and performed the MVCs. Thereafter, the participants started a 5-min warm-up at 100 W (90 rpm) followed by a 5-min rest and the 4000-m cycling TT. The same instructions and procedures given in the familiarization session were adopted during the experimental sessions.

During the warm-up and the time trial, the respiratory gas exchange was measured breath-by-breath to determine VO_2_ and VCO_2_. The PO was recorded every second (Tacx Flow T1680, Tacx, Wassenaar, Netherlands). During the MVC, and every 200-m during the time trial, the EMG of the right vastus lateralis (VL) was measured (Electromyography model 410C, EMGSystem Brazil, Sao Paulo, Brazil). The HR was measured with a heart rate transmitter coupled to the gas analyzer. The RPE was recorded every 1000-m using the Borg 15-point scale, ranging from “6” (no effort) to “20” (maximum effort) [25]. Twenty-five microliters of arterialized blood from the earlobe were collected at rest, immediately before (Pre-TT) and 1-min after (post-TT) the time trial to determine [La]. Samples were transferred to 1.5 ml micro tubes containing 25 µl of 1% sodium fluoride and immediately centrifuged at 3000 rpm at 4° C for ten minutes for plasma separation. Then, plasma lactate concentration was measured by colorimetric reactions using spectrophotometry (kit Biotecnica, Varginha, Brazil; Quimis, São Paulo, Brazil).

Prior to the MVC, the participants performed a standard warm-up consisting of four 5-s isometric contractions of the quadriceps muscles at an intensity corresponding to 50, 60, 70 and 80% of their subjective maximum; there was 30s of passive rest between repetitions [26]. Then, participants performed three 5-s MVCs of the quadriceps muscles interspersed by 1 min of passive rest (MVCpre). Individuals were verbally encouraged to exert maximum effort during each contraction. The quadriceps muscle strength of both legs was measured with a load cell (EMGSystem Brazil, Sao Jose dos Campos, Brazil) with the knee at an angle of 60° (full extension being considered 0°) and the hip at 90°. The MVCpre was established as the highest value recorded during the three MVC repetitions. An additional MVC was performed 2 min after the TT (MVCpost) and used to calculate the level of fatigue induced by TT. It was assessed via an index of fatigue expressed as the relative difference (%) between MVCpre and MVCpost.

Prior to the collection of electromyography signals, hair was removed by shaving, the skin lightly abraded to remove the outer layer of epidermal cells, and oil and dirt were removed from the skin with an alcohol swab to reduce skin impedance. A bipolar surface electrode Ag/AgCl (Hal, Sao Paulo, Brazil) was positioned over the VL muscle and the reference electrode in a neutral location (bone structure: tibia). Adhesive tape (Micropore TM 3M, Campinas, SP, Brazil) was used to fix the electrodes on to the skin. The placement and localization of the electrodes were in accordance with the recommendations of SENIAM [27]. The sampling frequency for acquisition of electromyography measurements was 2000 Hz (model 410c EMG System of Brazil Ltda, São Paulo, Brazil). The raw EMG signals were filtered with a Butterworth band pass filter with cut-off frequencies set at 10 and 400 Hz to remove movement artifacts and noise from external interference. The integrated EMG (iEMG) was calculated every 200 m. The iEMG obtained at every 200-m interval during the time trial was normalized by dividing the iEMG by the peak torque determined during the MVCpre. These procedures were performed using MATLAB 7.5 software (Mathworks Inc., Natick, US).

### Quantification of aerobic and anaerobic power

The aerobic and anaerobic contributions were calculated following the model adopted by Foster et al. [5]. First, the metabolic power (P_met_) during the warm-up was calculated using the following equation [3]:
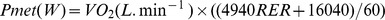



Where: P_met_ is the metabolic power; VO_2_ corresponds to oxygen uptake and RER the respiratory exchange ratio.

The gross mechanical efficiency was determined by dividing the warm-up power by P_met_. During the 4000-m TT, P_met_ was calculated every 200 m, assuming that the RER was equivalent to 1.00 [3]. The aerobic power output (P_aer_) was calculated every 200-m by multiplying the gross mechanical efficiency by P_met_. The anaerobic power output (P_an_) was obtained by subtracting the overall power output from P_aer_.

### Statistical analyses

Data distribution was analyzed using the Shapiro-Wilk test. The RPE, HR, [La], iEMG, PO, P_an_ and P_aer_ responses during the trials were compared using a two-way analysis of variance with repeated measures, with condition (CAF vs. PLA) and distance (200, 400, 600…4000-m) as factors. When necessary, subsequent post-hoc comparisons were made using Bonferroni correction. The paired Student’s t-test was used to compare the mean values of dependent variables (RPE, HR, [La], iEMG, PO, P_an_, P_aer,_ time, anaerobic, aerobic and total work) between the CAF and PLA conditions. The effect size (ES) and the 95% of confidence interval (95% CI) were calculated to verify caffeine effects on performance, as suggested by Conger et al. [28]. The Hedges correction (Hedges’s g) was used to account for potential bias resulting from the small sample size [18]. The ES of 0.2, 0.6 and 1.2 were considered as small, moderate, and large, respectively [29], [30]. Analyses were performed using SPSS (13.0) software, except for ES values, which were calculated in Comprehensive Meta analysis software. The smallest standardized change was assumed to be 0.20. Statistical significance was accepted at p<0.05.

## Results

Since all of the data were normally distributed in both conditions (p>0.05), parametric tests were used to identify statistically significant differences between CAF and PLA for all dependent variables.

### Mean power output and time

**Figure 1:**
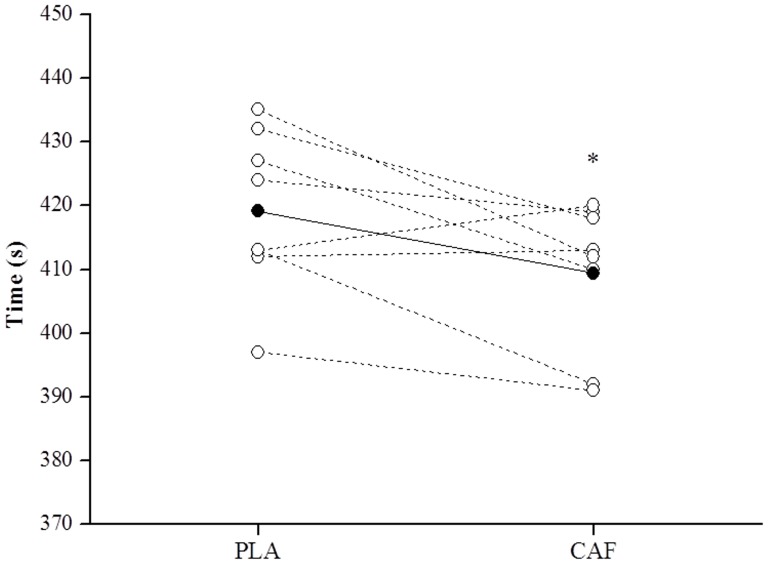
Time to complete a 4000-m cycling time trial after caffeine (CAF) or placebo (PLA) ingestion. Data are presented as mean (•) and individual (○) values (n = 8). * CAF was significantly faster than PLA.

The mean PO during the 4000-m cycling TT was significantly greater in the CAF than in the PLA condition [ES = 0.60 (95% CI = 0.04 to 1.16), p = 0.034] (Table 2). Although two participants did not improve their performance with caffeine ingestion (non-responders), on average, the time to complete the 4000-m TT was significantly faster in CAF than in PLA [409.4±11.6 vs. 419.1±12.6 s, respectively; ES = 0.71 (95% CI = 0.09 to 1.13), p = 0.026] (Fig. 1).

**Table 2:** Performance and physiological parameters during the 4000-m cycling time-trial in caffeine (CAF) and placebo (PLA) conditions.

	CAF	PLA
Power output (W)	232.8±21.4*	219.1±18.6
P_an_ (W)	64.9±20.1	57.3±17.5
P_aer_ (W)	167.9±4.3	161.8±11.2
Total work (J)	95245±8593	91789±7709
Anaerobic work (J)	26363±7361	23888±6795
Aerobic work (J)	68709±2118	67739±3912
VO_2_ (L.min^−1^)	4.01±0.10	3.87±0.26
iEMG (%MVC)	45.4±13.7	46.4±12.8
HR (bpm)	167±8	169±10
RPE (unit)	14±2	14±1

Values are means ± SD. Anaerobic power (P_an_), aerobic power (P_aer_), oxygen consumption (VO_2_), integrated electromyography (iEMG), maximal voluntary contraction (MVC), heart rate (HR) and rating of perceived exertion (RPE).*Significantly different from PLA (p<0.05).

**Figure 2:**
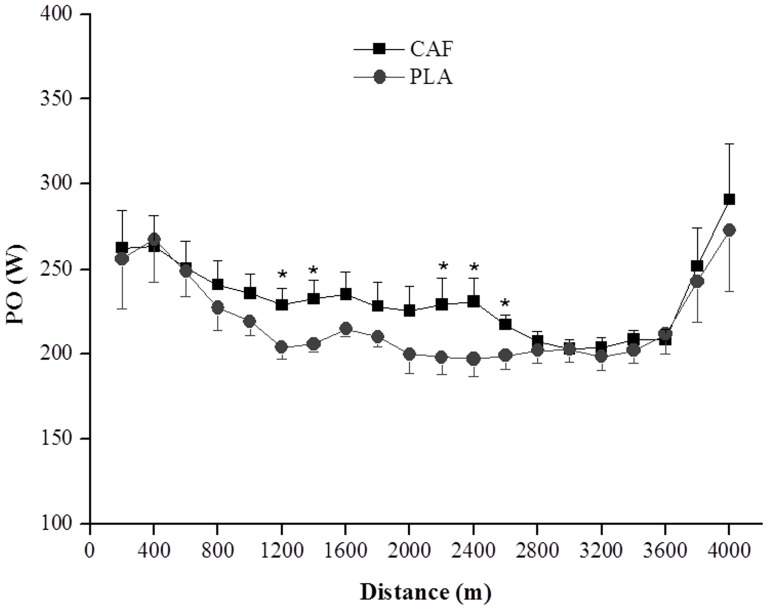
Power output for each 200-m cycling time trial after caffeine (CAF) and placebo (PLA) ingestion. Data are expressed as means ± SEM (n = 8). * CAF was significantly higher than PLA at 1200, 1400, 2200, 2400 and 2600 m.

Participants adopted a fast-start strategy in both the CAF and PLA conditions (p<0.05), but the PO remained elevated longer in CAF (Fig. 2). The PO at 1200, 1400, 2200, 2400, and 2600 m was significantly greater in the CAF than in the PLA (p<0.05). An end spurt was observed in both conditions, but was not significantly different between conditions.

### Aerobic and anaerobic power output

**Figure 3:**
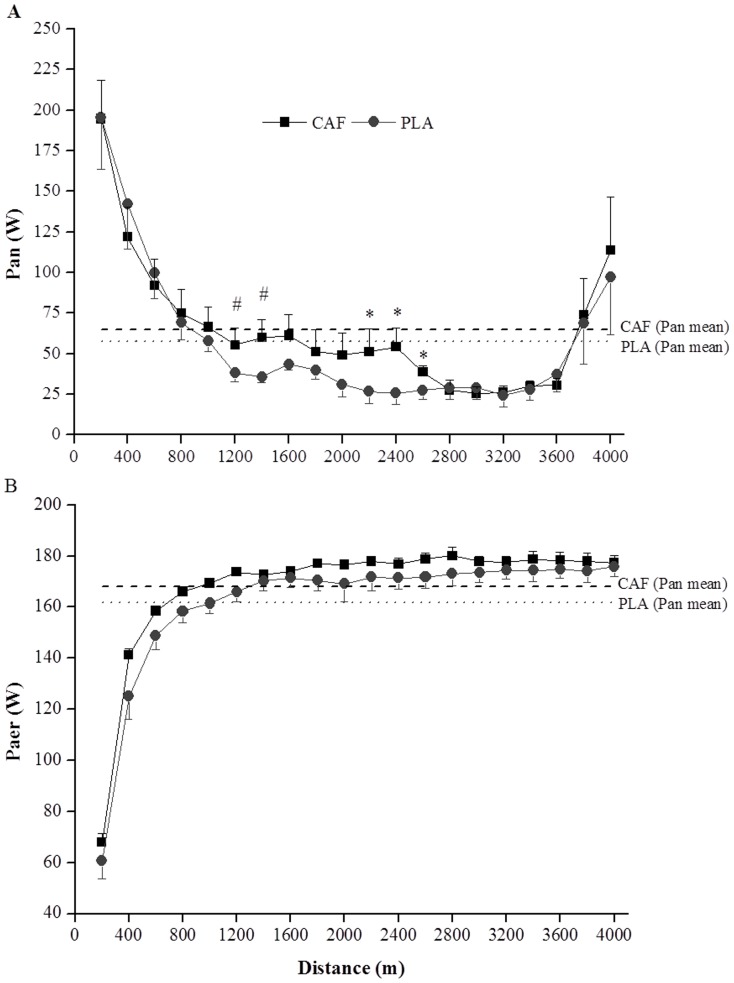
Mean and SEM values for anaerobic (P_an_, panel A) and aerobic (P_aer_, panel B) power output for each 200 m in the caffeine (CAF) and placebo (PLA) conditions (n = 8). * P_an_ was significantly higher in CAF than in PLA at the 2200-, 2400- and 2600-m intervals (p<0.05); # P_an_ tended to be greater in CAF than in PLA at 1200- and 1400-m intervals (p = 0.07).

**Figure 4:**
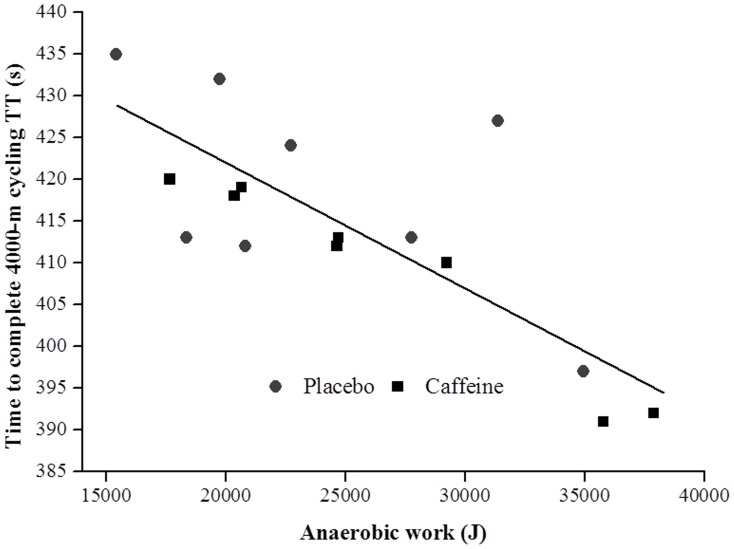
Relationship between time for the 4000-m cycling time trial and total anaerobic work for the caffeine and placebo conditions (n = 8). Pooled correlation coefficient was 0.77 (p<0.05).

The mean P_an_ and P_aer_ was not significantly different [ES = 0.35 (95% CI = −0.07 to 0.77), p = 0.103, and ES = 0.60 (95% CI = −0.21 to 1.40), p = 0.147, respectively] between CAF and PLA ingestion (Table 2). However, P_an_ at 2200, 2400 and 2600 m were higher (p<0.05) in CAF than in PLA (Fig. 3A). There was a tendency for the P_an_ values at 1200 and 1400 m to be higher in CAF than in PLA, but this did not reach statistical significance (p = 0.07). On the other hand, P_aer_ was not significantly different between the conditions (p>0.05) at any distance interval (Fig. 3B). No significant differences between CAF and PLA conditions were found for anaerobic, aerobic or combined aerobic and anaerobic work during the TT (Table 2). Time to complete the TT was negatively associated with total anaerobic work (*r* = −0.77, p<0.05; Fig. 4), and not associated with total aerobic work (*r* = 0.02, p = 0.93).

### Integrated electromyography

**Figure 5:**
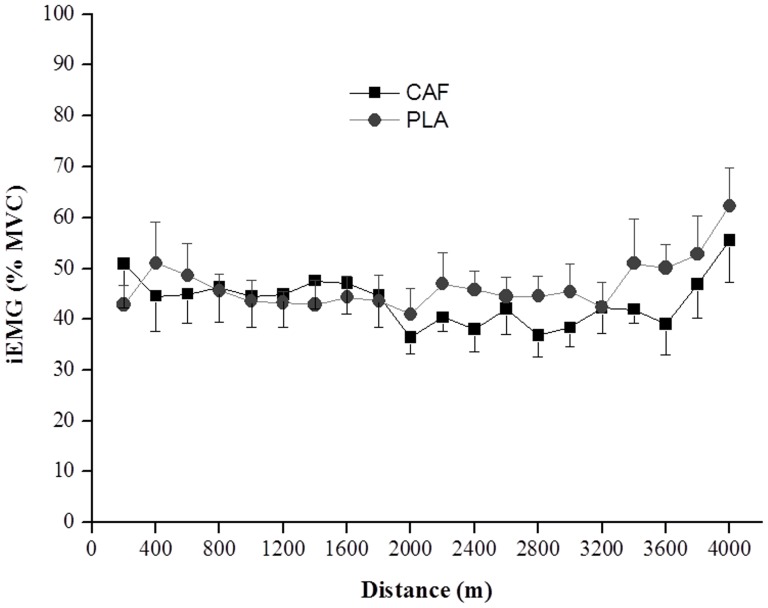
Mean and SEM values of integrated electromyography of the vastus lateralis expressed as a percentage of maximal voluntary contraction (MVC) every 200 m during the 4000-m cycling time trial in the caffeine (CAF) and placebo (PLA) conditions (n = 6).

There was no significant difference between CAF and PLA conditions for the average iEMG of the vastus lateralis during the trial (Table 2). In accordance, there were no significant differences between the conditions for any particular distance (Fig. 5). Two participants were excluded from all analyses of EMG data due to technical failure during the recording of the signal (n = 6).

### Heart rate, rating of perceived exertion, [La] and VO_2_

**Figure 6:**
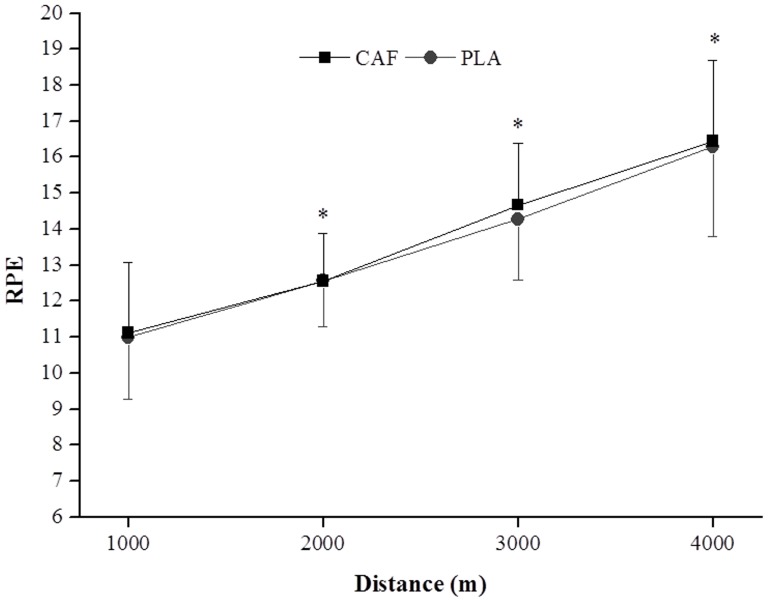
Mean and SEM values for rating of perceived exertion (RPE) every 1000 m during the 4000-m cycling time trial in the caffeine (CAF) and placebo (PLA) conditions (n = 7). * Significantly higher than all preceding values (P<0.05).

The HR increased during the first three intervals (200-, 400- and 600-m) in both conditions and thereafter remained constant throughout the test; there was no significant difference between the conditions. Similarly, the RPE increased progressively from 1000 m (PLA: 11.0±1.7 and CAF: 11.1±2.0 units) to 4000 m (PLA: 16.3±2.5 and CAF: 16.4±2.2 units) in both conditions, but there was no significant difference between them (Fig. 6). The [La] increased with exercise, but it was not significantly different (p>0.05) between conditions at rest (CAF: 1.5±0.7 vs. PLA: 1.3±0.7 mmol.L^−1^), pre-TT (CAF: 1.5±0.7 vs. PLA: 1.3±0.6 mmol.L^−1^), and post-TT (CAF: 9.7±1.6 vs. PLA: 9.0±2.5 mmol.L^−1^). Finally, the mean VO_2_ during the TT was similar between CAF and PLA conditions (Table 2).

### Index of fatigue

There was no significant difference (p>0.05) between CAF and PLA condition for the fatigue index (4.0±7.1 vs. 5.4±10.5%, respectively).

### Effect of order

There was no order effect (trial 1 versus trial 2) for any of the variables investigated (Table 3).

**Table 3:** Effect of order for performance and physiological parameters during the 4000-m cycling time-trial.

	TRIAL 1	TRIAL 2	P value
Power output (W)	225.4±26.3	226.5±14.2	0.894
P_an_ (W)	62.6±22.5	59.6±15.2	0.589
P_aer_ (W)	162.8±11.6	166.9±4.7	0.364
Total work (J)	93929±9802	93105±6604	0.755
Anaerobic work (J)	25743±8341	24508±5781	0.535
Aerobic work (J)	67599±3741	68849±2332	0.320
VO_2_(L.min^−1^)	3.9±0.3	4.0±0.1	0.367
iEMG (%MVC)	45.2±11.7	46.5±14.7	0.828
HR (bpm)	170±10	167±8	0.346
RPE (unit)	14±1	14±2	0.844

Values are means ± SD. Anaerobic power (P_an_), aerobic power (P_aer_), oxygen consumption (VO_2_), integrated electromyography (iEMG), maximal voluntary contraction (MVC), heart rate (HR) and rating of perceived exertion (RPE).

## Discussion

The main objective of the present study was to determine the impact of caffeine supplementation on performance, the distribution of both power output and anaerobic energy, and muscle recruitment during a 4000-m cycling time-trial. The main findings were: 1) a greater mean PO and lower final time during the TT when athletes ingested caffeine compared to PLA; 2) the PO in the middle of the TT (2200, 2400 and 2600 m) was greater in CAF versus PLA; 3) the higher PO values in the middle of the TT with caffeine ingestion were accompanied by a higher P_an_, but total anaerobic work remained unchanged, although it was correlated with time to complete the TT; 4) there was no alteration in iEMG signal during any part of the trial. To the best of our knowledge, the present study is the first to demonstrate that caffeine ingestion alters pacing strategy, anaerobic contribution and performance during a short-distance cycling TT.

Although we have found that two participants were non-responders to caffeine, both the mean PO and time to complete the trial were improved (∼10 s faster, moderate ES = 0.71, P<0.05) after ingestion of caffeine (5 mg.kg^−1^ body mass). In addition, the mean improvement with caffeine ingestion was increased slightly when the two non-responders are not taken in account (∼14 s faster). We did not identify any order effect for the variables investigated, suggesting that the results cannot be attributed to learning effect or something other than the effects of caffeine. This is in accordance with the findings of Wiles et al. [17], who found an improvement in mean PO and a lower final time after caffeine ingestion in well-trained cyclists during a 1-km cycling TT. These results also corroborate with a reduction in final time to complete a longer TT (30-min TT, ∼70% of the maximum power output) after caffeine ingestion [31]. However, the mechanisms by which caffeine increased the performance during the TT were not explored in any of these studies.

The ergogenic effects of caffeine can be explained by a stimulating effect on the CNS and/or by a direct action on skeletal muscle [32]. In the CNS, caffeine is a bioactive molecule that stimulates neuron activity as it easily crosses the blood-brain barrier due to its lipophilic properties [33]. There is some evidence suggesting that caffeine at physiologic, nontoxic concentrations exerts an ergogenic effect centrally by inhibiting adenosine receptors [14]. Adenosine is an endogenous neuromodulator that decreases excitatory neurotransmitter release, reducing the firing rates of central neurons [34]. Caffeine ingestion has also been associated with a reduction in pain perception [13] and a lower RPE [11], probably via a hyperalgesic effect promoted by blockade of adenosine A_2_a receptors [14]. In the present study, although mean PO was higher in CAF than PLA, the RPE was not significantly different between the conditions, suggesting that participants were able to perform the TT with a higher PO/RPE ratio with caffeine ingestion. This result is in accordance with other studies showing that caffeine increases the PO/RPE ratio during a given TT [15], [16].

Even with a higher PO, the iEMG signal was not different between conditions, suggesting caffeine may have improved peripheral muscle function during the exercise. It has been suggested that iEMG may not be interpreted uniquely as a muscle activation parameter, since there is the possibility that changes in iEMG activity are the result of altered motor neuron firing rates mediated either centrally [35] or peripherally as a response to a reduction in muscle relaxation time and contraction speed [36]. Nonetheless, during dynamic exercise, changes in iEMG amplitude have been the only way to indirectly measure muscle activation levels [37], and there is some evidence supporting that changes in iEMG signal may reflect change in muscle activation during controlled-experimental conditions as in our case [38], [39]. Thus, it seems reasonable to hypothesise that in the present study caffeine may have exerted its main ergogenic effects by reducing RPE for a given PO, and improving muscle function, with no evidence of a significant effect on muscle activation (as indicated by iEMG). In addition, although the average power output during the TT was higher in CAF than in PLA, there was no significant difference in the fatigue index between the conditions. This is consistent with previous research [40] and suggest that caffeine was able to induce a greater power output during the trial without inducing any additional fatigue at the end.

It has been suggested that the main peripheral effects of caffeine are: 1) an increase in the activity of the Na^+^ and K^+^ pump [41]; 2) an increase in calcium mobilization from the sarcoplasmic reticulum [42] and; 3) an increase in glycolysis via a direct effect on PFK [9]. Furthermore, the inhibition of the phosphodiesterase (PDE) enzyme in the muscle results in elevated levels of intracellular cAMP, which exerts control on the major kinases stimulating glycogenolysis [43]. Although we cannot fully disregard any of these mechanisms, it seems unlikely that the caffeine dose ingested in the present study would have affected calcium mobilization since this effect has only been demonstrated *in vitro* when toxic doses of caffeine are utilized [14]. Additionally, an increase in calcium mobilization and Na^+^ and K^+^ pump activity would have increased the excitability of the muscle fibers, which should have altered iEMG activity. Instead, we found no changes in iEMG activity with caffeine throughout the trial. In contrast, caffeine increased the P_an_ in the middle of the trial, suggesting that the improved power output during the time-trial may have been supported by an additional anaerobic energy supply related to any peripheral alterations provoked by caffeine action.

Although caffeine increased P_an_ in the middle of the trial, there were no significant differences between CAF and PLA conditions for total anaerobic or aerobic work during the TT. It has previously been reported that the amount of anaerobic energy that can be produced during a TT is a constant value, independent of pacing strategy [3]. As a consequence, it would be expected that P_an_ at the beginning or the end of the trial would be reduced in the caffeine TT to compensate for the greater P_an_ in the middle of TT. Instead, we observed that P_an_ at the beginning and the end of the TT was similar between the two conditions. However, even though it was not statistically significant, caffeine intake was associated with a total anaerobic work ∼10% higher than placebo, and total anaerobic work was significantly correlated with time to complete the TT. An increase of 10% in the total anaerobic work is compatible with results from previous studies that reported a similar increase (10%) in the anaerobic capacity (measured by MAOD) during supramaximal, time-to-exhaustion exercise after caffeine intake [7], [8], but it is larger than the 2.5–4% variation in the total anaerobic work described after different induced pacing strategies [3], [4]. It could be hypothesized from these results that caffeine may have allowed the use of a small additional "anaerobic reserve" (∼10%) that is not used under normal conditions. The existence of this anaerobic reserve during a TT has recently been demonstrated by Corbett et al. [44], who found that the total anaerobic energy yield during a 2000-m cycling TT was higher when participants believed they were competing against another athlete of similar ability (head-to-head), than when they exercised alone (time trial), suggesting that a motivational stimulus promotes the use of a greater degree of the anaerobic ‘‘reserve’’. Thus, a small increase in the total anaerobic work found in the present study after caffeine ingestion, probably via an effect associated with increased muscle glycogenolysis and glycolysis, contributed to an improvement in the overall performance.

Concerning the pacing strategy adopted, the athletes adopted a fast-start in both conditions, but were able to maintain a greater PO during the middle of the trial (1200-, 1400-, 2200-, 2400- and 2600-m intervals) in CAF versus PLA. The changes in the PO were mirrored by similar changes in the P_an_, corroborating the idea that power distribution along a TT appears to be regulated primarily by changes in the anaerobic contribution [2]–[4]. It is interesting to note that caffeine intake was able to increase anaerobic contribution only during the middle of the trial. To the best of our knowledge, there is no study investigating the effect of caffeine on pacing strategy and anaerobic distribution during a TT. However, we [45], [46] and others [47] have shown that metabolic (e.g., muscle glycogen depletion), performance level or psychological (e.g., listening to music) manipulation are able to alter pacing strategy within minutes of starting a TT. Rauch et al [47], manipulating initial muscle glycogen reserves, reported that participants started two identical time trials (1-h TT) at almost the same workload (∼230 W), but after 1min of cycling the workload was ∼10 W higher and averaged 14 W higher throughout the carbohydrate-loaded diet compared with the normal diet TT. In the present study, we found that pacing strategy with caffeine intake started to change after 1.2 km (∼1.5 min) compared with the placebo TT. These results suggest that the pacing strategy at the beginning of a given TT may be regulated by a feed-forward, anticipatory mechanism, based on pre-exercise expectations and experiences, but it may be influenced by peripheral feedback as the exercise progresses [48]. In addition, although PO and P_an_ were increased in the middle of the trial, there were no differences beyond 2600 m. The PO and P_an_ during the end-spurt were similar for both the CAF and PLA conditions, and were accompanied by a similar post-exercise [La], suggesting that CAF was not able to increase anaerobic contribution at the end of the trial. However, a similar end spurt after having produced greater power throughout the middle portion of the TT is an interesting and meaningful outcome. It would be expected that a greater power output in the middle would result in greater failure or attenuated ability to produce an end spurt. Instead, it is plausible to suggest that the influence of caffeine also appears in the end spurt enabling participants to produce a similar sprint finish even after producing more power in the middle portion.

A potential limitation of the present study is that circulating caffeine concentrations were not measured. However, the participants were asked not to consume caffeine in the 24 h prior to each experimental test. Adherence to this advice was controlled via diet records, which indicated that participants followed the recommendations. In addition, it has been demonstrated that the ingestion of 5 mg.kg^−1^ body mass of caffeine one hour before the main trial, as adopted in the present study, is sufficient to significantly raise caffeine plasma concentration [9], [40], [49], [50]. While measures of circulating caffeine concentration would have confirmed, especially in the PLA condition, that no diet caffeine was consumed prior the trial, participants were assigned to the two conditions using a randomized, double-blinded, random and counterbalanced design, and we found an improvement in the TT performance after caffeine ingestion compared to placebo. This provides indirect evidence that plasma caffeine levels were higher in CAF than in PLA.

## Conclusion

In conclusion, the results of the present study suggest that athletes were able to complete a 4000-m cycling TT more quickly when ingesting 5 mg.kg^−1^ of caffeine, compared with a placebo. The improvement in the performance with caffeine intake resulted from a greater anaerobic energy contribution in the middle of the trial, whereas the aerobic energy contribution and total anaerobic energy expenditure were not significantly different.
